# Development and applications of a monoclonal antibody against caprine interferon-gamma

**DOI:** 10.1186/s12896-019-0596-5

**Published:** 2019-12-23

**Authors:** Wen-Tao Ma, Qi Liu, Meng-Xia Ning, Yu-Xu Qi, Saad Rehman, De-Kun Chen

**Affiliations:** College of Veterinary Medicine, Northwest A&F University, Yangling, 712100 Shaanxi Province China

**Keywords:** Prokaryotic expression, Caprine interferon-gamma, Monoclonal antibody, Contagious ecthyma, Immunofluorescence

## Abstract

**Background:**

Interferon-gamma (IFN-γ) is an important mediator of type I immune response and has antiviral, immunoregulatory and anti-tumor properties, plays a wide range of roles in inflammation and autoimmune diseases. The aim of this study was to obtain monoclonal antibody (mAb) against caprine IFN-γ by immunizing of BALB/c mice with the purified rIFN-γ.

**Results:**

Recombinant caprine IFN-γ was expressed in *Escherichia coli* strain BL21 (DE3) and monoclonal antibodies against caprine IFN-γ were produced by immunizing of BALB/c mice with rIFN-γ. One hybridoma secreting mAb was screened by enzyme-linked immunosorbent assay (ELISA) which was designated as 2C. MAb secreted by this cell line were analyzed through ELISA, western blot and application of the mAb was evaluated by immunofluorescence analysis using goat lip tissues infected with Orf virus. ELISA analysis revealed that mAb 2C can specifically recognize rIFN-γ protein and culture supernatant of goat peripheral blood mononuclear cells (PBMCs) stimulated by concanavalin A (Con A) but cannot recognize the fusion tag protein of pET-32a. Western blot analysis showed that mAb 2C can specifically react with the purified 34.9 kDa rIFN-γ protein but does not react with the fusion tag protein of pET-32a. Immunofluorescence results demonstrated that mAb 2C can detect IFN-γ secreted in histopathological sites of goats infected with Orf virus.

**Conclusions:**

A caprine IFN-γ-specific mAb was successfully developed in this study. Further analyses showed that the mAb can be used to detect IFN-γ expression level during contagious ecthyma in goats.

## Background

IFN-γ is a critical cytokine for innate and adaptive immunity that plays a wide range of roles in inflammation and autoimmune diseases [1, 2]. IFN-γ is an important mediator of type I immune response and has antiviral, immunoregulatory and anti-tumor properties [3, 4]. In general, IFN-γ can exert antiviral function by binding to its receptor directly [5] and can also promote pathogen killing by activating macrophages [6]. In addition, IFN-γ also possesses potent immunomodulatory capacities [7]. IFN-γ can stimulate macrophages and T lymphocytes to express class II MHC molecules, thus enhancing their antigen-presenting capacity [8]. Aberrant IFN-γ expression can be initially determined when the host was invaded by external pathogens [9]. Therefore, the level of IFN-γ can be used as an early diagnostic indicator of diseases to assess the body’s immune level and health state [10].

Contagious ecthyma is an infectious disease which is caused by Orf virus that primarily occurs in sheep and goat but infects humans as well [11]. The disease is widely distributed around the world and causes huge economic losses [12]. Orf virus mainly infects lambs by forming erythema, pustules and scars on the eyelids, lips, nares and feet, which seriously affects the sucking of lambs and causes weight loss [13, 14]. For decades, contagious ecthyma has been a major problem and constrained the development of the small ruminant dairy industry [15]. Previous studies have shown that Orf virus can stimulate host cells to express certain antiviral proteins (e.g. IFN resistance protein) [16], which can destroy or inhibit viral infection of the host [17]. In addition, IFN-γ can improve the host immune function and inflammatory responses to Orf virus [18]. Cytokines, including IFN-γ, produced following T cell activation in response to pathogen infection can be used for disease diagnosis [19]. Anderson et al. in 2001 have detected differential IFN-γ mRNA expression by cells in primary versus reinfection skin lesions during the course of Orf virus infection in sheep. They found that IFN-γ mRNA expression was significantly increased after reinfection, which was closely related to the host’s resistance to Orf virus infection [20]. These findings suggest that the level of IFN-γ in the host can be used as a sensitive indicator to evaluate the immune function of the host after infection with Orf virus. Therefore, detecting the level of IFN-γ is essential for assessing the immune status of the host.

In this study, we immunized BALB/c mice with prokaryotic expressed rIFN-γ protein and obtained hybridoma cells 2C that specifically recognize caprine IFN-γ. MAb 2C can be used to detect the IFN-γ expression level of goat infected with Orf virus by immunofluorescence. Our study provides great convenience for early diagnosis of contagious ecthyma and lays a foundation for antiviral mechanism investigation of IFN-γ. Furthermore, the mAb can also serve as a useful tool for IFN-γ diagnostic kits and colloidal gold test strips of goats.

## Results

### Analysis of the expression of IFN-γ from PBMCs of goats infected with Orf virus using real-time PCR

**Fig. 1:**
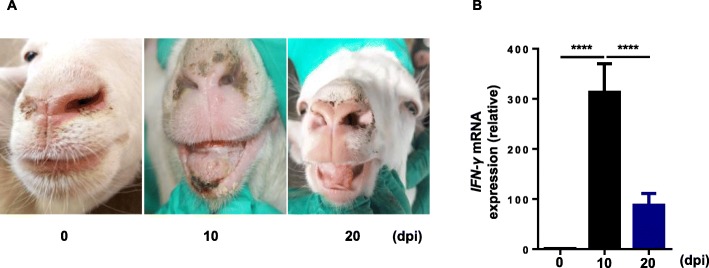
Analysis of the expression of IFN-γ from PBMCs of goats infected with Orf virus using real-time PCR. **a** Pathological changes in the lip of the goat on 0, 10th and 20th days post infection (dpi) with Orf virus. **b** The relative expression of IFN-γ of goat PBMCs on 0, 10th and 20th dpi with Orf virus (*n* = 8)

Goat blood was collected on 0, 10th and 20th days after infection with Orf virus. Lymphocytes were isolated and RNA was extracted. Then the expression of IFN-γ cytokine-encoding mRNA was analyzed by real-time PCR. The results showed that at the 10 days post infection (dpi) with Orf virus, there were pustules and scars on the lips of goats (Fig. 1a). Meanwhile, real-time PCR analysis indicated that relative expression of IFN-γ mRNA was significantly higher at 10 dpi (Fig. 1b). At 20 dpi, the scars on goat’s lips disappeared (Fig. 1a) and accompanied by a decrease in the relative expression of IFN-γ (Fig. 1b). These results indicated that IFN-γ played an important role in controlling the severity of the disease during Orf development.

### Expression, purification and SDS-PAGE analysis of rIFN-γ

**Fig. 2:**
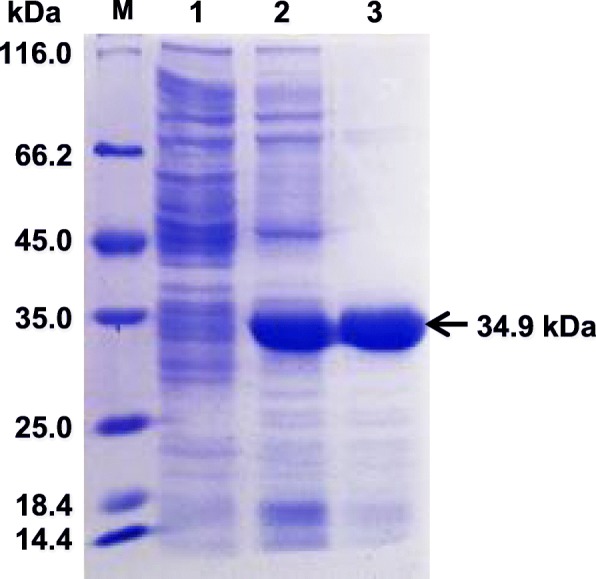
Expression, purification and SDS-PAGE analysis of caprine rIFN-γ. The caprine rIFN-γ was expressed in *E. coli* strain BL21 and the proteins were visualized by Coomassie brilliant blue R-250. Lane 1: marker, lane 2: rIFN-γ before induction, lane 3: induced rIFN-γ by IPTG, lane 4: purified rIFN-γ; Arrow: the induced and purified rIFN-γ of 34.9 kDa

The *E. coli* strain BL21 that contained recombinant plasmid pET-32a caprine IFN-γ was cultured in LB medium and the expression of rIFN-γ was induced by IPTG. SDS-PAGE showed that induced protein band was enhanced at 34.9 kDa (Fig. 2, lane 2). The rIFN-γ was purified by Ni-NTA agarose. SDS-PAGE indicated that purified protein was 34.9 kDa and protein band was single. Furthermore, no other protein was shown (Fig. 2, lane 3), which could be used for the preparation of mAb.

### Generation and characterization of mAb against rIFN-γ

**Fig. 3:**
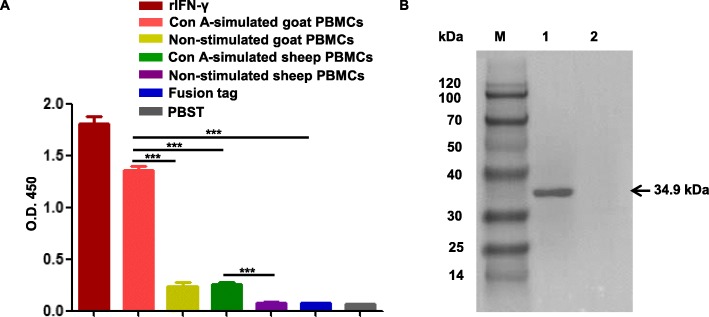
**a** Generation and specificity determination of mAb 2C by ELISA. For the ELISA analysis, purified rIFN-γ protein (1 μg), fusion tag protein of pET-32a (1 μg), Con A-stimulated or non-stimulated goat or sheep PBMCs were served as antigens. In addition, the culture supernatant of 2C hybridoma cells was served as the primary antibody (*n* = 6, ***P < 0.001). **b** Western blot analysis of reactivity of mAb 2C. M, protein marker; lane 1, rIFN-γ protein; lane 2, fusion tags protein of pET-32a

Mice were immunized for 4 times with rIFN-γ and rIFN-γ protein was used as antigen for hybridoma screening by ELISA. The results showed that the 2C mAb specifically recognized rIFN-γ and PBMCs culture supernatant stimulated by Con A but didn’t recognize recombinant tag fusion protein of PET 32a (fusion tag) (Fig. 3a). Furthermore, we performed western blot analysis using rIFN-γ and fusion tag protein. The results showed that the 2C mAb reacted with rIFN-γ but didn’t exhibit reactivity against fusion tag protein (Fig. 3b). This result indicated that 2C mAb specifically recognized rIFN-γ protein and native IFN-γ but did not recognize fusion tag protein.

### Immunofluorescence analysis of frozen sections of lip tissues from Orf virus-infected goats

**Fig. 4:**
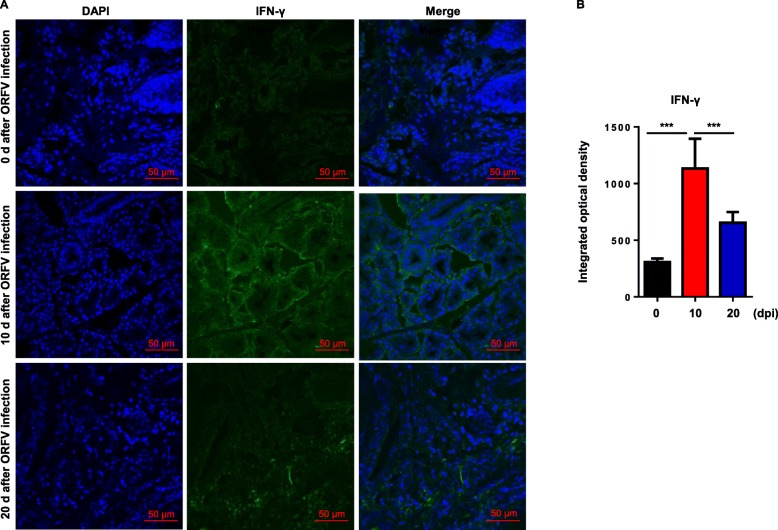
Analysis of IFN-γ expression level in lip tissues of Orf virus-infected goats using immunofluorescence staining. **a** IFN-γ (green) expression level was analyzed by immunofluorescence staining in Orf virus-infected tissues at various days after infection. Cell nuclei were stained with DAPI (blue). **b** Integrated optical density analysis of immunofluorescence staining results (*n* = 8, ***P < 0.001).

Previous studies have shown that mononuclear cells and neutrophils accumulation can be detected in the lip skin of the lamb after infection with Orf virus and IFN-γ plays a prominent role in host immune responses that control disease severity [20]. Therefore, the level of IFN-γ in histopathological tissues can be used as an important indicator to evaluate the immune function of body and the severity of disease. Therefore, we selected frozen sections of goat lip tissues after infecting with Orf virus for immunofluorescence analysis. The result showed that there was a large amount of IFN-γ secretion in the lip tissues of goat at 10 dpi. During the course of development and recovery of the disease, the amount of IFN-γ secretion decreased on the 20 dpi with Orf virus (Fig. 4a). Integrated optical density analysis showed the same result (Fig. 4b). The mock result was presented in Additional file 1: Figure S1. These results were consistent with Fig. 1, as it showed that IFN-γ was closely related to the severity of the disease in terms of both mRNA level and protein level.

## Discussion

Type I and type II interferons play important roles in the cellular immune response against viral infection. The immunomodulatory effects of IFN-γ are more prominent, which is more conducive for the establishment of long-term antiviral status [5, 21]. Interferon undergoes signal transduction and produces a wide range of enzymes, transcription factors and cytokines to exert a broad spectrum of antiviral effects [22]. Thus, the greater the amount of IFN-γ secreted means a stronger cellular immune function of the body against viruses [10]. In the present study, goat IFN-γ monoclonal antibodies were used to detect the secretion of IFN-γ in the pathological sites at different periods after infection with Orf virus. The results showed that the secretion of IFN-γ in the lesion was closely related to the severity of clinical symptoms (Fig. 1 and Fig. 4). It precisely reflects the host’s antiviral status and serves as a basis for disease diagnosis.

In addition to its direct antiviral effects, IFN-γ also exerts antiviral effects indirectly by regulating other immune responses. IFN-γ can activate macrophages and monocytes, which can selectively kill many bacteria and tumor cells [23]. IFN-γ can also enhance Fc receptor-mediated phagocytosis and the promotion effect is maximized within 6–8 h after serum IFN-γ level reaches the peak [24, 25]. Therefore, IFN-γ can be used as an important indicator of immunity to reflect the immune status of the body and assess the immune effect of the vaccine.

Currently, the assessment of IFN-γ level is mostly achieved by real-time PCR, which is performed based on the transcription level of the messenger RNA. However, the biological function of IFN-γ is achieved at the protein level. It is generally known that the production of a protein requires two important processes, namely transcription and translation [26, 27]. Obviously, there is a delay between transcriptional induction and protein production. In addition, post-transcriptional regulation and post-translational modification could affect the final state of a protein. Therefore, in many scenarios, transcript levels are not sufficient to predict protein levels. In this study, we immunized BALB/c mice with prokaryotic expressed rIFN-γ and screened a hybridoma cell line that specifically recognized goat IFN-γ by indirect ELISA. The hybridoma cells can recognize rIFN-γ as well as nature IFN-γ but do not recognize the fusion tag protein and have excellent specificity. This antibody can be applied in ELISA, western blot and immunohistochemistry and can accurately detect the secretion level of goat IFN-γ at the protein level. Thus, the antibody developed in the present study provides a useful tool for assessing the immune status of goats and for studying the host’s anti-infection immunity.

In conclusion, the direct application of the goat IFN-γ monoclonal antibody prepared in this study can serve for early diagnosis of goat diseases. Furthermore, it will be helpful in the development of control strategies and disease prevention. In addition, the monoclonal antibody can be utilized for antibody labeling technology and to establish an immunoassay method, which provides great convenience for the study of the pathogenic mechanism of certain diseases of goats and the antiviral infection mechanism of IFN-γ.

## Conclusions

In this study, a monoclonal antibody named 2C can specifically recognize caprine IFN-γ was successfully prepared by immunizing mice with caprine rIFN-γ protein. It can be used to detect IFN-γ secretion of different periods of goat infected with Orf virus.

## Methods

### Animals

Female BALB/c mice were purchased from the laboratory animal center of the Air Force Medical University, Xi’an city, Shaanxi Province, China. All mice were bred in the animal center of NWAFU in specific pathogen-free facilities and treated in accordance with the guidelines of the Care and Use of Laboratory Animals of the Ministry of Health, China. The mice were euthanized by spine dislocation during mAb collection. Eight healthy 2-month-old goats were purchased from a farm in Yangling city. During sample collection, goats were anesthetized with sodium pentobarbital (Sigma-Aldrich, St. Louis, Missouri, USA) at a dose of 20 mg/kg intravenously. After the study, all goats were fully recovered and normally fed in the animal center of NWAFU.

### Analysis of the expression of IFN-γ from goat PBMCs infected with Orf virus using real-time PCR

Goat blood was collected on 0, 10th, and 20th day after infection with Orf virus and lymphocytes were separated by lymphocyte separation solution (Haoyang, Tianjin, China). RNAiso plus was added (Takara Bio, Beijing, China) to save at − 80 °C. Total RNA extraction was done by using the following methodology: First, take above samples out of − 80 °C and add 1/5 volume of chloroform after they melt. To mix the solution properly, vigorously shake for 15 s. Then centrifuge at 12000 rpm for 15 min at 4 °C and pipette the upper aqueous phase into a new Eppendorf tube. Second, add the equal volume of isopropanol, mix and leave it at room temperature for 10 min. Then centrifuge at 12000 rpm for 10 min at 4 °C and discard the supernatant. Third, add 1 ml of 75% DEPC ethanol to wash RNA and centrifuge at 12000 rpm for 10 min at 4 °C. After the centrifugation discard the supernatant. Finally, add 10 to 100 μL of RNase-free double distilled water to dissolve the RNA for reverse transcription. The RNA reverse-transcription was done with the help of a Fastking RT kit (Tiangen, Beijing, China). Real-time PCR was performed with a SYBR Green Real-time PCR kit (Tiangen) and the mRNA expression of IFN-γ was normalized to that of β-actin. The sequence of caprine IFN-γ was obtained from GenBank (NM_001285682.1) and the primers was designed with Primer Permier 5.0 software. The primer sequences are AGATCCAGCGCAAAGCCATA (forward) and TCTCCGGCCTCGA AAGAGAT (reverse). Each test set up 3 technical repeats.

### Preparation of natural and recombinant caprine IFN-γ

Natural caprine IFN-γ could be produced by spleen cells and PBMCs [28]. In the present study, the coding sequence region of caprine IFN-γ (signal peptide sequence removed) was cloned from Con A (Sigma-Aldrich) activated PBMCs and then inserted into the expression vector PET 32a, and the recombinant vector was transformed into the host cell BL21 (DE3) to construct a recombinant expression bacteria named “cIFN-γ-PET 32a-BL21 (DE3)”. The recombinant expression bacteria “cIFN-γ-PET 32a-BL21 (DE3)” was cultured in ampicillin LB medium to logarithmic phase at 37 °C. After that, 0.03 mmol/L isopropyl-D thiogalactopyranoside (IPTG) (Sigma-Aldrich) was added to the bacterium solution to induce IFN-γ expression for 6 h at 30 °C. Bacteria were harvested by centrifugation at 6000 g for 30 min at 4 °C and pellets were resuspended in 100 ml of phosphate buffer (PBS). Then, bacteria were lysed by sonication on ice and the supernatant was collected by centrifuging at 12000 rpm for 30 min. Expression levels of IFN-γ proteins in both supernatant and precipitates were analyzed by sodium dodecyl sulfate polyacrylamide gel electrophoresis (SDS-PAGE).

The prokaryotically expressed recombinant fusion protein caprine IFN-γ (rIFN-γ) protein was purified from the supernatant under native conditions. The Ni-NTA agarose (Solarbio, Beijing, China) was used to purify recombinant caprine IFN-γ protein according to the manufacturer’s instructions. In brief, the supernatant was added to the Ni-NTA agarose and incubated for 3 h at 4 °C. Then, the heterologous proteins were eluted with 40 mmol/L imidazole (Solarbio) and the target proteins were eluted with 400 mmol/L imidazole. The concentration of purified protein was determined by using the BCA protein concentration assay kit (Biosharp, Shanghai China) according to the manufacturer’s instructions.

### Animal immunization and hybridoma lines production

Animal immunization was conducted as previously reported [29]. Briefly, six-to-eight-week-old female BALB/c mice were immunized intraperitoneally with 100 μg of purified rIFN-γ protein for three times with an interval of 21 days. The first injection was accompanied with complete Freund’s adjuvant (Sigma-Aldrich), the second and third injections were accompanied with incomplete Freund’s adjuvant (Sigma-Aldrich). For the fourth immunization, 100 μg of rIFN-γ protein solution (pH = 7.4) was injected through a caudal vein. Three days after the last boost injection, splenocytes were harvested from the mice and fused with SP2/0 myeloma cells (10:1 ratio) to establish hybridoma lines, using polyethylene glycol 1500 (Roche, Basel, Schweizer) as fusogen. The fused cells were resuspended in RPMI 1640 medium (Thermo Fisher, Shanghai, China) supplemented with 10% FBS (ExCell Bio, Shanghai, China), 100 units/ml penicillin and streptomycin (Hayao, Haerbin, China), and HAT media supplement (Sigma-Aldrich). They were then plated into 96-well tissue culture plates and cultured at 37 °C with 5% CO_2_. After incubation for 7 to 10 days, the culture medium in each well was analyzed by indirect ELISA to detect the presence of mAb against rIFN-γ. Positive hybridoma cells were subjected to limiting dilution three times. Finally, one hybridoma line 2C was selected for further investigation.

### Indirect enzyme-linked immunosorbent assay

The mAb screened by indirect ELISA was performed as follows: 96-well polystyrene microtiter plates (Corning, New York, USA) were coated with purified rIFN-γ protein (100 μL/well, 10 mg/ml) and incubated overnight at 4 °C. The plates were washed three times by PBST (PBS containing 0.05% tween-20) and blocked with 0.1 M carbonate buffer containing 5% skimmed milk powder. After washed as above, cultured supernatant from the hybridomas was added to each well and incubated for 1 h at 37 °C. Followed by previous step, 100 μL of HRP-conjugated goat-anti-mouse IgG (Bioss, Beijing, China) diluted in 1:5000 with PBST was added as secondary antibody and incubated for 1 h at 37 °C. After washing, 100 μL of 3,3′,5,5′-tetramethylbenzidine (Tiangen) was added and incubated for 18 min at 37 °C. After chromogenic termination by 0.2 M H_2_SO_4_, absorbencies were measured with an automatic ELISA reader (Bio-Rad, California, USA) at 450 nm.

### Preparation of mice ascites

For this, 10-week-old female BALB/c mice were selected and received sterile liquid paraffin with 0.5 ml by intraperitoneal injection. After 7 to 10 days, the cultured hybridoma cells were collected by centrifugation at 800 rpm for 10 min and 10^6^ cells in 0.5 mL were injected into mice intraperitoneally. After 10 days, the abdominal circumference of the mice increased significantly and the ascites were collected. The fluid from the mouse ascites was centrifuged and stored at − 20 °C.

### SDS-PAGE and Western blot

To identify the specificity of mAb, SDS-PAGE and western blot techniques were applied. For western blot assay, rIFN-γ and some unrelated proteins were respectively mixed with SDS (Sigma-Aldrich) sample buffer supplied with β-mercaptoethanol (Sigma-Aldrich) and denatured for 10 min at 100 °C. First, the mixtures were separated by SDS-PAGE and then transferred to polyvinylidene difluoride membranes (TianGen) under 60 V for 50 min using transfer buffer (58 mM Glycine, 71.8 mM Tris base, 1.9 mM SDS). After the membranes were blocked with 3.5% fish gelatin in PBST at 37 °C for 2.5 h and washed three times with TBST, hybridoma culture supernatant was served as the primary antibody and incubated overnight at 4 °C. Followed by previous step, HRP-conjugated goat-anti-mouse IgG (Sigma-Aldrich) was served as the secondary antibody incubated at 25 °C for 1 h. Finally, the signal was visualized with enhanced chemiluminescence reagent ECL and ChemiDocTMMP Imaging System (Bio-Rad).

### Immunofluorescence assay

To identify whether mAb recognize IFN-γ secreted by histopathological sites, frozen sections of lip tissues of goat infected with Orf virus were used for immunofluorescence analysis. First, frozen sections were rewarmed with an antigen-repairing solution and incubated at room temperature (RT) for 15 min. After being washed six times with PBS (pH = 7.4), 5% Triton-100 was added and incubated at RT for 15 min to permeabilize the cells. Followed by washing, frozen sections were blocked with PBS containing 5% donkey serum for 2 h at RT. Then, hybridoma culture supernatant was served as primary antibody and incubated for 1 h at 37 °C. Followed by previous step, fluorescein isothiocyanate (FITC)-conjugated goat-anti-mouse IgG (Proteintech Group, Chicago, USA) was diluted 400 times with PBS as secondary antibody at RT for 2 h in the dark. Nuclei were stained with DAPI (BioFROXX, Guangzhou, China) for 30 min at RT in dark. Corresponding tissue section of healthy goat was served as negative control. Finally, sections were analyzed using an inverted fluorescence microscope (Leica, Wetzlar, Germany).

### Statistics

The statistical analysis was performed using the two-tailed Students’ *t* test. Differences were considered statistically significant if *p* < 0.05 (* *p* < 0.05, ** *p* < 0.01, *** *p* < 0.001).

## Supplementary information

**Additional file 1: Figure S1.** Negative control for immunofluorescence staining results of Orf virus-infected lip tissues. (A) Immunofluorescence staining results of tissues treated with PBS and DAPI (*n*=8).

## Data Availability

The datasets used or analyzed during the current study are available from the corresponding author on reasonable request.

## Abbreviations

Con A: Concanavalin A
dpi: days post infection
ELISA: Enzyme-linked immunosorbent assay
IFN-γ: Interferon-gamma
IPTG: Isopropyl-β-d-thiogalactoside
mAb: monoclonal antibody
PBMCs: Peripheral blood mononuclear cells
PCR: Polymerase chain reaction
rIFN-γ: recombinant interferon-gamma
RT: Room temperature
SDS-PAGE: Sodium dodecyl sulfate-polyacrylamide gel electrophoresis
