# Supplementary material for: Development and applications of a monoclonal antibody against caprine interferon-gamma
Source: BMC Biotechnol. 2019 Dec 23;19:102. doi: 10.1186/s12896-019-0596-5 (PMC6929374; doi:10.1186/s12896-019-0596-5)
Supplement: Supplementary file 1 — Additional file 1: Figure S1. Negative control for immunofluorescence staining results of Orf virus-infected lip tissues. (A) Immunofluorescence staining results of tissues treated with PBS and DAPI (n=8). [file 12896_2019_596_MOESM1_ESM.pptx]

## Slide 1
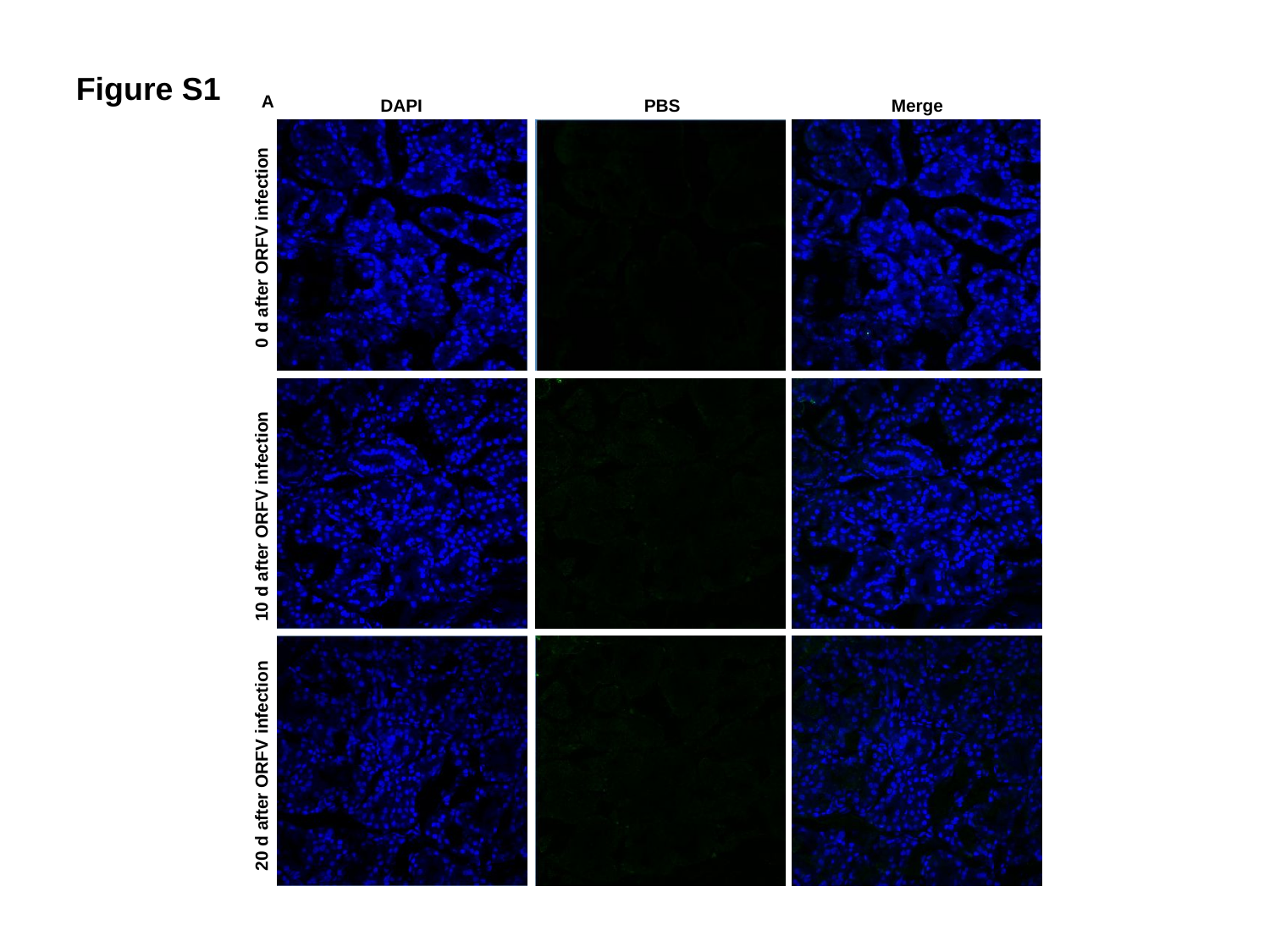

Figure S1
A
DAPI
PBS
Merge
0 d after ORFV infection
10 d after ORFV infection
20 d after ORFV infection
